# Rugose Morphotype in *Salmonella* Typhimurium and *Salmonella* Heidelberg Induced by Sequential Exposure to Subinhibitory Sodium Hypochlorite Aids in Biofilm Tolerance to Lethal Sodium Hypochlorite on Polystyrene and Stainless Steel Surfaces

**DOI:** 10.3389/fmicb.2019.02704

**Published:** 2019-11-27

**Authors:** Mohit Bansal, Ramakrishna Nannapaneni, Divya Kode, Sam Chang, Chander S. Sharma, Christopher McDaniel, Aaron Kiess

**Affiliations:** ^1^Department of Poultry Science, Mississippi State University, Mississippi State, MS, United States; ^2^Department of Food Science, Nutrition and Health Promotion, Mississippi State University, Mississippi State, MS, United States

**Keywords:** *Salmonella*, sodium hypochlorite, chlorine, rugose development, food contact surfaces, biofilm, confocal scanning microscopy, transmission electron microscopy

## Abstract

*Salmonella* biofilms act as a continuous source for cross-contamination in the food processing environments. In this study, a stable rugose morphotype of *Salmonella* was first induced by sequential exposure to subinhibitory concentrations (SICs) of sodium hypochlorite (NaOCl) (ranging from 50 to 300 ppm over 18-day period) in tryptic soy broth. Then, rugose and smooth morphotypes of *Salmonella* Typhimurium ATCC 14028 and *Salmonella* Heidelberg ATCC 8326 were characterized for biofilm forming abilities on polystyrene and stainless steel surfaces. Rugose morphotype of both ATCC 14028 and ATCC 8326 exhibited higher Exopolysaccharide (EPS) formation than smooth morphotype (*p* ≤ 0.05). Also, the SICs of NaOCl (200 or 300 ppm in broth model) increased the biofilm formation ability of rugose morphotype of ATCC 8326 (*p* ≤ 0.05) but decreased that of ATCC 14028. The 2-day-old *Salmonella* biofilms were treated with biocidal concentrations of 50, 100, or 200 ppm NaOCl (pH 6.15) in water for 5, 10, or 20 min at room temperature. The biofilm reduction in CFU/cm^2^ for the rugose was lower than the smooth morphotype on both surfaces (*p* ≤ 0.05) by lethal NaOCl in water. Scanning electron micrographs on both polystyrene and stainless steel surfaces demonstrated that the rugose morphotype produced a denser biofilm than the smooth morphotype. Transmission electron micrographs revealed the cell wall roughness in rugose morphotype, which may help in tolerance to NaOCl. The gene expression data indicate that the expression of biofilm regulator (*csgD*), curli (*csgA, csgB*, and *csgC*), and cellulose (*bcsE*) was significantly increased in rugose morphotype when induced by sequential exposure of NaOCl SICs. These findings reveal that the rugose morphotype of *S.* Typhimurium and *S.* Heidelberg produced significantly denser biofilm on food contact surfaces, which also increased with sequential exposure to SICs of NaOCl in the case of *S.* Heidelberg, and these biofilms were more tolerant to biocidal NaOCl concentrations commonly used in the food processing plants.

## Introduction

Every year, foodborne salmonellosis caused by non-typhoidal *Salmonella* is responsible for 380 deaths and 19,000 hospitalizations in the USA (Scallan et al., 2011). Often *Salmonella* forms biofilms on different food contact surfaces (Stepanovic et al., 2004). The biofilms are aggregates of bacterial colonies living in organized structures and act as a continuous source of cross-contamination (Davey and O’Toole, 2000). Biofilm formation is associated with the expression of flagella, fimbriae, pili, curli, and extra polymeric compounds [EPC(s)] such as proteins, DNA, and polysaccharides (Palmer et al., 2007; Flemming and Wingender, 2010; Steenackers et al., 2012).

In the food processing facilities, disinfectants such as sodium hypochlorite (NaOCl), quaternary ammonium compounds (QAC), and organic acids such as peroxyacetic acid and acetic acid are frequently used (Russell and McDonnell, 2000). Previous findings show that the gradual exposure to sublethal concentration of antimicrobials (including disinfectants) induces bacterial adaptation that leads to the development of antimicrobial tolerant phenotypes of foodborne bacterial pathogens (Abeysundara et al., 2016; Bansal et al., 2018).

Morphological adaptation for bacteria is a kind of biological function, which aids in the development of tolerance to various stresses. These morphological adaptations occur in multiple species under certain conditions. Nutrient deprivation or low temperature exposure induces viable but not culturable cells (VBNCs), which are associated with morphological adaptation (van Teeseling et al., 2017). Liu et al., 2017 observed that VBNCs of *Escherichia coli* are in the shorter rod shaped form. Previous study shows the ability of *Salmonella* to switch to rugose morphotype in tryptic soy agar within 3 days at 25°C (Anriany et al., 2001). Rugosity in *Salmonella* is a multicellular behavior, which is manifested as aggregation of cells in an extracellular matrix within the micro-colonies (Anriany et al., 2001). This morphotype development is observed as rough dry and red (rdar) colonies on agar cultures. Rugose morphotype is also demonstrated as firm pellicle formation at air-liquid interphase and biofilm formation on the solid abiotic surfaces (Obe et al., 2018).

Previous studies show that environmental stress and metabolic status such as nutrient depletion, microaerophilic conditions, temperature, and 4% of ethanol determine the multicellular behavior of *Salmonella* (Gerstel and Römling, 2003). Curli (*csgBAC* operon) and cellulose (bacterial cellulose synthesis operons) are the two major components of *Salmonella* biofilm. *CsgD* regulates the biofilm synthesis by activating the transcription of curli and cellulose synthesis genes (White et al., 2006). The transcription of *csgD* is regulated by *ompR*, integration host factor (IHF), and histone-like nucleoid structuring (H-NS) proteins in response to environmental cues and metabolic stress. Other genes such as *spiA* (Dong et al., 2011), *csrA* (Potts et al., 2019) flagellar synthesis, and motility (*fimA, fimH*, and *fliC*) (Kolenda et al., 2019) also play role in the surface attachment of cells and biofilm formation.

Rugose morphotype of *Salmonella* and *Vibrio cholerae* confers tolerance to their planktonic cells against NaOCl (Yildiz and Schoolnik, 1999; White et al., 2006). Similarly, the pellicle rugose cells of *Salmonella* were found to be significantly less susceptible to lethal exposure of NaOCl (Scher et al., 2005). Also, biofilm confers higher tolerance to antimicrobials compared to planktonic bacteria. Previous studies reported that the extra polysaccharide compounds of rugose planktonic cells provide tolerance against desiccation (Gibson et al., 2006), osmotic and oxidative stress (Anriany et al., 2001). The surrounding matrix of rugose morphotype may help in the formation of NaOCl tolerant biofilms on different food contact surfaces, which could be a survival mechanism for *Salmonella* in stressful environmental conditions.

Bacterial biofilm formation is influenced by many biotic (strains, growth phase, and metabolic activity) and abiotic factor (temperature, pH, and nutrient availability) (Davey and O’Toole, 2000; Chmielewski and Frank, 2003). These factors may influence any of the different steps involved in biofilm formation such as cell motility, EPC(s) production, and quorum sensing (cell to cell signaling) (Singh et al., 2017). Previous studies reported that reactive oxygen species can suppress quorum sensing process and environmental cues can modulate EPC(s) production, cell motility, and surface proteins by controlling the concentration of second messengers such as cAMP and c-di-GMP (Kavanaugh and Horswill, 2016). Dhakal et al., 2019 observed that factors such as temperature, nutrient availability, type of surface, and exposure to sublethal NaOCl may influence *Salmonella* smooth morphotype biofilm formation ability.

In our previous study, we reported that the adaptation of *Salmonella* to sublethal NaOCl induced rugose morphotype (Obe et al., 2018). However, the rugose morphotype produced by sublethal NaOCl was not further characterized and the inactivation of such rugose biofilms by lethal NaOCl was not studied. Also, there is a knowledge gap on the biofilm architecture of rugose morphotype induced by subinhibitory NaOCl and their gene expression data. Therefore, the focus of this study is as follows: (1) to further characterize the sublethal NaOCl-induced *Salmonella* rugose morphotype biofilm formation by *Salmonella* Typhimurium ATCC 14028 and *Salmonella* Heidelberg ATCC 8326; (2) to determine the efficacy of lethal NaOCl in water for inactivating such rugose biofilms on two different food contact surfaces, polystyrene and stainless steel; and (3) to determine the biofilm- and rugose-related gene expression in *S.* Typhimurium ATCC 14028 induced by sublethal NaOCl.

## Materials and Methods

### Bacterial Strains and Culture Preparation

Two *Salmonella* serotypes, *S.* Typhimurium ATCC 14028 and *S.* Heidelberg ATCC 8326, were used in the present study. These strains were stored as a stock culture in tryptic soy broth (TSB; Sigma-Aldrich Co., St. Louis, MO) supplemented with 25% glycerol at −80°C. Before experiments, cells from frozen stock were streaked on tryptic soy agar (TSA; Sigma-Aldrich Co.) plates and incubated for 24 h at 37°C. Later, a subculture was obtained after inoculating a single colony into 10 ml of TSB and incubation at 37°C for 24 h.

### Chlorine Source

NaOCl solution (5%, AC4149552500, ACROS Organics, Morristown, NJ) was used as a stock solution for available chlorine. This stock was stored in dark and at 4°C. Fresh solution of NaOCl concentrations of 200, 300, 400, 500, and 600 ppm in TSB for MIC assay and the 50, 100, and 200 ppm of NaOCl concentrations in sterile water for biofilm inactivation assay were prepared using the 5% stock NaOCl solution before each experiment.

### Determination of Minimum Inhibitory Concentration and Subinhibitory Concentrations of Sodium Hypochlorite in Tryptic Soy Broth

The determination of minimum inhibitory concentration (MIC) and subinhibitory concentration (SIC) of NaOCl were determined using the macrodilution method in TSB as per CLSI, 2016. To determine MIC, *Salmonella* cell concentration of 6 log CFU/ml was obtained from serial 1/10 dilution of smooth morphotype grown at 37°C for ~22 h, followed by addition of different NaOCl concentrations (200, 300, 400, 500, and 600 ppm prepared from 5% NaOCl stock) in 15 ml polypropylene tubes. Thereafter, the tubes were incubated for 24 h at 37°C. The lowest concentration of NaOCl in TSB, which inhibited the visible turbidity after 24 h as per CLSI 2016, was determined as MIC, while below the MIC concentrations, which did not inhibit bacterial growth, were selected as SIC.

### Development of *Salmonella* Rugose Morphotype by Subinhibitory Sodium Hypochlorite

Smooth morphotype of both *Salmonella* serotypes were exposed to increasing NaOCl concentration to induce rugosity development. The inoculum of 10^6^ cells/ml was sub-cultured with 50 ppm of NaOCl in TSB for 3 consecutive days at 37°C for 24 h. Thereafter, a similar subculture pattern was followed to induce stress in 50 ppm NaOCl increments every 3 days to a maximum concentration of 300 ppm (18 days of stress). Thereafter, the rugose morphotype (rough, dry, and filamentous) was exhibited on TSA at 37°C after 24 h. For control cells, *Salmonella* smooth cells were subcultured in a similar manner but without NaOCl exposure in TSB. The stability of rugose morphotype was determined after subculturing cells in TSB and TSA without NaOCl. Rugose cells developed characteristic corrugated, dry and rough morphology on TSA after 24 h at 37°C. Later, rugose cells were grown overnight in TSB at 37°C and harvested by centrifugation. A log of 10^9^ cells/ml were stored as frozen in 25% glycerol stock at −80°C for further experiments. The MIC for both morphotypes (smooth and rugose) of both *Salmonella* serotypes for NaOCl was determined using the macrodilution method in TSB as previously described. Further, to observe difference in rugose and smooth colony formation, an aliquot of bacterial load (10^3^ CFU/ml) of both the morphotypes was plated on LB agar containing Congo red (40 mg/L) and coomassie brilliant blue (20 mg/L). The plate was incubated at 37°C for up to 48 h. Thereafter, images of *Salmonella* smooth and rugose morphotype colony edge pictures were acquired and analyzed by EVOS XL Core inverted digital microscope with 4X objective.

### Biofilm Formation by Rugose and Smooth Morphotypes on Polystyrene Surface by Crystal Violet Assay

Biofilm formation of rugose and smooth morphotypes was measured by crystal violet assay (O’Toole, 2011). An overnight culture of smooth and rugose morphotype was grown in TSB and was diluted to achieve a final inoculum level of ∼10^6^ CFU/ml. Biofilm was developed after inoculation of 200 μl TSB culture containing 6 log CFU/ml of bacteria in individual wells of a 96 well polystyrene microtiter plate (SKU 229190, Celltreat Scientific Product, Pepperell, MA) and incubation at 25°C for 48 h. After 48 h of incubation, bacterial inoculum was aspirated out from each well of a plate. Thereafter, the wells were washed five times with sterile distilled water to remove all loosely attached cells. The plate was air dried for 45 min and biofilm was stained with 250 μl of 0.41% w/v crystal violet dye (AC447570500, ACROS organics) for 45 min. The dye was pipetted out from individual wells and to remove excess of dye, wells were washed five times with sterile distilled water. Plates were air dried for 45 min and thereafter, stained wells were destained with 95% ethyl alcohol. The OD_600_ was measured using a micro-quant microplate spectrophotometer (Model ELx800, BioTek Instruments, Winooski, VT). The experiment was replicated four times with eight replicate wells used for each treatment.

### Biofilm Formation by Rugose and Smooth Morphotypes on Polystyrene and Stainless Steel Surfaces by CFU Enumeration Method

CFU enumeration method was used for quantification of smooth and rugose morphotype biofilm cells attached on polystyrene and stainless steel surface for each strain independently. Like crystal violet method, an aliquot of 200 μl of TSB containing ∼10^6^ CFU/ml was added into individual well of a 96-well polystyrene microtiter plate (SKU 229190, Celltreat Scientific Product, Pepperell, MA) in duplicate. Plate was incubated for 48 h at 25°C. Each well was aspirated, and loose or unbound cells were removed by washing wells three times with sterile PBS. An aliquot 200 μl of 0.1% buffered peptone water was added into individual wells and strongly attached biofilm cells were removed with sterile cotton swab method (Desai et al., 2012; Soni et al., 2013). The content of the well along with the cotton swab was placed in a 1.5 ml Eppendorf tube filled with 800 μl of 0.1% buffered peptone water. The tubes were vortexed vigorously for 2 min to remove cells from the cotton swab, first dilution was obtained by transferring 100 μl of initial culture to 900 μl of 0.1% buffered peptone water, followed by 1/10 serial dilution procedure and 100 μl of aliquots were plated on duplicate TSA plates and incubated for 24 h at 37°C for CFU enumeration.

Stainless steel (SS) coupons (#4 finish, 1.5 cm × 1 cm, Stainless Supply, Monroe, NC) were used for biofilm formation and enumeration. SS coupons were autoclaved at 121°C for 15 min prior to use. The SS coupons were immersed completely in individual wells of 24 well plates with 2 ml of *Salmonella* 6 log CFU/ml culture/well. Plate was incubated for 48 h at 25°C. Each well was aspirated, and each coupon was washed three times with sterile PBS to remove loosely or unbound cells. Coupons were placed in 15 ml polypropylene tubes with five glass beads of diameter, 5 mm (VWR international GA, USA) and 5 ml of 0.1% buffered peptone water (Soni et al., 2013; Konrat et al., 2016). Tubes were vortexed vigorously for 2 min, first dilution was obtained by transferring 200 μl of initial culture to 800 μl of 0.1% buffered peptone water, followed by 1/10 serial dilution procedure for spread plating and colonies were enumerated on duplicate TSA plates after 24 h of incubation at 37°C.

### Quantification of Exopolysaccharide Production by Ruthenium Red Staining

Exopolysaccharide (EPS) formation by rugose and smooth morphotypes of each strain was measured by ruthenium red assay (Borucki et al., 2003; Upadhyay et al., 2013). Overnight incubated rugose and smooth cells in TSB were harvested by centrifugation at 5,000 ×*g* for 10 min at 4°C. The obtained pellet was resuspended in fresh TSB and serially diluted to obtain a cell concentration of 10^6^ CFU/ml. Thereafter, cells (200 μl) were transferred to each one of the eight wells of a 96-well microtiter plate. The plate was incubated for 48 h at 25°C and loose or unattached cells were removed by pipetting out the bacterial inoculum. Each well of the plate was washed three times with sterile PBS to remove loosely or unbound cells. Plate was air dried at room temperature for 45 min and wells were added with 250 μl of ruthenium red (0.1%) stain solution. The biofilm was stained for 45 min at room temperature. Wells without *Salmonella* biofilms were also added with 250 μl of ruthenium red (0.1%) stain solution to act as blanks. After staining, the residual ruthenium red stain solution from each well was carefully transferred to individual wells in a new polystyrene 96-well microtiter plate. The EPS production was measured by subtracting the OD_600_ of a blank well (OD_B_) value to the OD_600_ value of the residual stain (OD_S_) collected from sample wells respectively. The OD_600_ was measured using a micro-quant microplate spectrophotometer (Model ELx800, BioTek Instruments, Winooski, VT). The experiment was replicated four times with eight replicate wells used for each treatment.

### Effect of Subinhibitory Sodium Hypochlorite on Rugose and Smooth Morphotype Biofilm Formation in Broth

The *Salmonella* biofilm formation of each strain was obtained in a 96-well microtiter plate in the presence of subinhibitory NaOCl of 200 or 300 ppm in TSB (treatment) or in the absence of NaOCl exposure (control) at 25°C for 48 h. The organic content of TSB interacts with NaOCl and reduces its bioavailability. Thus, the NaOCl concentrations of 200 or 300 ppm were used in TSB. Biofilm formation after subinhibitory NaOCl exposure was measured by crystal violet assay (O’Toole, 2011) as described before in the Section “Biofilm Formation by Rugose and Smooth Morphotypes on Polystyrene Surface by Crystal Violet Assay.” The experiment was replicated four times with eight replicate wells used for each treatment.

### Inactivation of Rugose and Smooth Morphotype Biofilm Using Sodium Hypochlorite on Polystyrene Surface in Sterile Water

Sodium hypochlorite inactivation efficiency for rugose and smooth morphotype of each strain of *Salmonella* biofilm was studied using three different exposure times and NaOCl concentration levels in sterile water. An initial inoculum level of 6 log CFU/ml of 200 μl of stationary phase culture was added into individual wells of a 96-well polystyrene microtiter plate. *Salmonella* biofilm formation was developed at 25°C for 48 h. After development of biofilm, loose or unbound cells were pipetted out and wells were washed three times with sterile PBS to remove loosely attached cells. Each biofilm well was exposed to concentrations of 0, 50, 100, and 200 ppm NaOCl (pH 6.15) in sterile water for 5, 10, or 20 min at room temperature. The pH 6.15 was adjusted with 1 N HCl in these aqueous NaOCl solutions. While NaOCl of 200–300 ppm was considered sublethal concentration in broth, but even 50 ppm was found to be biocidal in water since there is no organic content to neutralize NaOCl. After chlorine exposure, NaOCl solution was pipetted out from individual wells and they were washed with 250 μl of sterile PBS three times to remove any remnant of NaOCl. An aliquot of 200 μl of 0.1% buffered peptone water was added in each well and cells were quantified following removal from the well surface with a sterile cotton swab (Desai et al., 2012; Soni et al., 2013). Biofilm cells on polystyrene surface which survived after inactivation assay were quantified as discussed before. CFU counts were used to quantify rugose and smooth morphotype biofilms after NaOCl inactivation and to distinguish factors such as morphotype variation, exposure time and concentrations. The minimum detection limit by this method was 10 CFU/polystyrene well. The experiment was replicated three times.

### Inactivation of Rugose and Smooth Morphotype Biofilm Using Sodium Hypochlorite on Stainless Steel Surface in Sterile Water

The biofilm of rugose and smooth morphotype of each strain of *Salmonella* was developed on SS coupons (No. 304 and finish 4) at 25°C for 48 h. The SS coupons were immersed completely in individual wells of 24-well plates with 2 ml of *Salmonella* 6 log CFU/ml culture/well. After biofilm formation, planktonic cells were removed by pipetting out and coupons were washed three times with sterile PBS to remove loosely attached cells. Each biofilm on coupons was exposed to 0, 50, 100 and 200 ppm NaOCl (pH 6.15) concentration for 5, 10, or 20 min. Biofilm cells on SS surfaces, which survived after inactivation assay were quantified as discussed before. The minimum detection limit by this method was 50 CFU/SS coupon. The experiment was replicated four times.

### RNA Isolation and Real Time Quantitative PCR

Rugose and smooth morphotypes of *S.* Typhimurium ATCC 14028 was grown to early stationary phase in TSB at 37°C for 15 h. The total RNA was extracted using TRIzol as described before (Rio et al., 2010) and cDNA was prepared using M-MLV (NE Biolab). The mRNA levels of rugose morphotype and biofilm-associated genes were determined using SYBR Green PCR Master mix (Bio-Rad) on a Bio-Rad 384-well Real Time PCR System. The primers for each gene (Table 1) were designed using published GenBank ST sequences using NCBI Primer-BLAST tool. Data were normalized to endogenous control (16S rRNA). The relative gene expression was determined using the comparative critical threshold (Ct) value method and level of targeted gene expression between smooth (control) and rugose (treatment) morphotypes was compared and expressed as fold change in gene expression to investigate the effect of subinhibitory concentration of sodium hypochlorite on the development of rugose morphotype.

**Table 1:** List of primers used in this study.

Gene with accession number	Primer	Sequence (5′__3′)
16S-rRNA (NC_003198.1)	Forward	5′-GGCGCATACAAAGAGAAGCG-3′
	Reverse	5′-CTCCAATCCGGACTACGACG-3′
*envZ* (NC_003198.1)	Forward	5′-CTCTCTCGCGACAAGCTGAT-3′
	Reverse	5′-CGTCCGGTACGAAGACGTAG-3′
*spiA* (NC_003198.1)	Forward	5′-AAGTCACCACAGATGGCTGG-3′
	Reverse	5′-GGGTGGCAAGAAAATTGCGT-3′
*bcsE* (NC_003198.1)	Forward	5′-GAGCGATACTCGCCCATCAA-3′
	Reverse	5′-TTGCCCCACGATTTGCTTTG-3′
*csgD* (NC_003197.2)	Forward	5′-GTGAGTAATGCGGACTCGGT-3′
	Reverse	5′-GTGAGTAATGCGGACTCGGT-3′
*ycfR* (NC_003197.2)	Forward	5′-TCAACAGAAGTTCGGCACCA-3′
	Reverse	5′-ATACGGAAAGACTTCGCCCC-3′
*csgA* (NC_003197.2)	Forward	5′-AACGACCATTACCCAGAGCG-3′
	Reverse	5′-TTACCGCCGTATTGACCGAC-3′
*csgB* (NC_003197.2)	Forward	5′-GGATTGCAACCGCGACAAAT-3′
	Reverse	5′-GGCACTATTATCCGTGCCGA-3′
*csgC* (NC_003197.2)	Forward	5′-TATTGCTCCTTGCCGCACTT-3′
	Reverse	5′-ACCTGAGGGATCACCGTGTA-3′
*hns* (NC_003197.2)	Forward	5′-TGCGCAGGCAAGAGAATGTA-3′
	Reverse	5′-ACGAGTGCGTTCTTCCACTT-3′
*rpoS* (NC_003197.2)	Forward	5′-GTTTGGTTCATGATCGCCCG-3′
	Reverse	5′-GCTTATCCGTGCAGTCGAGA-3′
*ompR* (NC_003197.2)	Forward	5′-TTCGTTTGCCTGACGACGTA-3′
	Reverse	5′-GCGAAGGGTGAAGAGGTTGA-3′
*ihf* (NC_003197.2)	Forward	5′-GCGGTTTCGGCAGTTTTTCT-3′
	Reverse	5′-ACTTTATCGCCGGTCTTGGG-3′
*fimA* (NC_003197.2)	Forward	5′-GCGTGAGTGGCGGTACTATT-3′
	Reverse	5′-GTACGGTATTGACCCAGCGT-3′
*fimH* (NC_003197.2)	Forward	5′-ATCCGTCCGGCGTCATAAAA-3′
	Reverse	5′-CGCCGTCCCGTTTGAATTAC-3′
*fliC* (NC_003197.2)	Forward	5′-ACGGTTTCTCACCGTAACCC-3′
	Reverse	5′-GGCTATTTCGCCGCCTAAGA-3′
*csrA* (NC_003197.2)	Forward	5′-AGCCTGGATACGCTGGTAGA-3′
	Reverse	5′-TTTAGGGGTGAAGGGCAACC-3′

### Confocal Scanning Microscopy of Rugose and Smooth Biofilms

To observe the three-dimensional distributions of cellular and extracellular components of both morphotypes of both *Salmonella* ATCC 14028 and ATCC 8326 strains, confocal laser scanning microscopy was performed using a Zeiss LSM 510 microscope with an oil immersion lens using 63X magnification. Biofilm was formed at 25°C in TSB using the Lab-Tech four- chamber no. 1 borosilicate glass coverslip system (Lab-tek, Nalge Nunc International, Rochester, NY). Biofilm was fixed using 1/2 strength Karnowsky’s fixative (pH 7.2). Fixed biofilms were stained with 250 μg/ml alexa flour 647 conjugate, 10 μM syto 9 and 10x sypro red (ThermoFisher Scientific, Grand Island, NY USA) stains successively for 30 min each to identify formation of different components of biofilm such as EPS, nucleic acid and proteins, respectively. Fluorochromes were excited using a kryptoneargon mixed-gas laser with a PMT2 filter. Randomly selected four different fields were analyzed to observe the biofilm formation of rugose and smooth morphotype.

### Scanning Electron Microscopy of Rugose and Smooth Morphotype Biofilms

Rugose and smooth morphotype biofilm morphology was also studied by using scanning electron microscopy. *Salmonella* biofilm was developed on Nunc Thermanox polystyrene cover slips (MA, USA) and the SS coupons at 25°C for 48 h. Coverslips and coupons were washed three times with sterile saline to remove loosely attached cells and fixed in 1/2 strength Karnowsky’s fixative (pH 7.2) overnight at 4°C. Both coverslip and coupons were washed three times with sterile distilled water and post fixed in 2% buffered (0.1 M sodium cacodylate) osmium tetroxide, followed by dehydration through a graded ethanol series [35, 50, (2X) 70, (2X) 95, and (4X) 100]. The coverslips and coupons were later dried using a critical point dryer (Autosamdri®-931, Tousimis) and sputter-coated with platinum (20 nm). Thereafter, coverslips were analyzed on a scanning electron microscope (JEOL JSM-6500F Field Emission Scanning Electron Microscope, MA, USA) to obtain micrographs. Four randomly selected areas were analyzed to study rugose and smooth morphotype biofilm formation.

### Transmission Electron Microscopy of Rugose and Smooth Cells

Ultrastructural morphology of rugose and smooth morphotype of *S.* Typhimurium (ATCC 14028) was prepared for transmission electron microscopy (TEM) by previously described methods (Capita et al., 2014). Overnight incubated rugose and smooth cells in TSB were harvested by centrifugation at 5,000 ×*g* for 10 min at 4°C and the resulting concentrated cell pellets were prepared for TEM. Pellets were fixed using 1/2 strength Karnovsky fixative in 0.1 M sodium cacodylate buffer (pH 7.2) overnight at 4°C. Fixed cells were washed in buffer, post fixed in 2% buffered osmium tetroxide, dehydrated through a graded ethanol series, and embedded in Spurr’s resin. Ultra-thin sections were cut using a Riecher Jung Ultra cut microtome, collected on copper grids and stained with uranyl acetate and lead citrate and viewed on a JEOL JSM-1230 (Jeol USA, MA, USA) at 80 kv. Four randomly selected areas were analyzed to study the ultrastructure changes in rugose and smooth morphotype.

### Statistical Analysis

The crystal violet biofilm measurement assay and ruthenium red EPS measurement assay were replicated four times and OD_600_ values were analyzed in a 2 (strain) × 2 (morphotype) factorial arrangement of treatments in a randomized complete block design. Biofilm measurement by CFU enumeration by plate counting was replicated three times for each strain independently and CFU/cm^2^ values were analyzed in 2 (surface) × 2 (morphotype) factorial arrangement of treatments in a randomized complete block design. The biofilm formation in the presence of subinhibitory NaOCl experiment was replicated independently for each strain four times and OD_600_ values were analyzed in a 2 (morphotype) × 4 (NaOCl concentrations) factorial arrangement of treatments in a randomized complete block design. The biofilm inactivation experiment was replicated independently for each strain (three times on polystyrene and four times on SS) and log-transformed counts were analyzed in a 2 (morphotypes) × 4 (NaOCl concentrations) × 3 (incubation times) factorial arrangement of treatments in a randomized complete block design. The means were separated using Fisher’s protected LSD when *p* ≤ 0.05. The gene expression data was analyzed by using Student’s *t*-test for the comparisons between smooth and rugose morphotypes. The data was analyzed using Statistical Analysis Software (SAS V 9.4) (SAS Institute, Cary, NC, USA).

## Results

### Determination of Minimum Inhibitory Concentration and Subinhibitory Concentration of Sodium Hypochlorite to *Salmonella* Typhimurium and *Salmonella* Heidelberg

The MICs of NaOCl for *S*. Typhimurium ATCC 14028 and *S*. Heidelberg ATCC 8326 smooth morphotype was 400 ppm, while it was 500 ppm for rugose morphotype in TSB. Results showed that higher concentration of NaOCl was required to inhibit planktonic rugose morphotype growth in TSB. Regardless of these differences between morphotypes, the SICs used were 200 and 300 ppm of NaOCl in TSB for both smooth and rugose of ATCC 14028 and ATCC 8326.

### Sequential Exposure to Subinhibitory Concentrations of Sodium Hypochlorite Induced Stable Rugose Morphotype Formation in *Salmonella* Typhimurium and *Salmonella* Heidelberg

**Figure 1:**
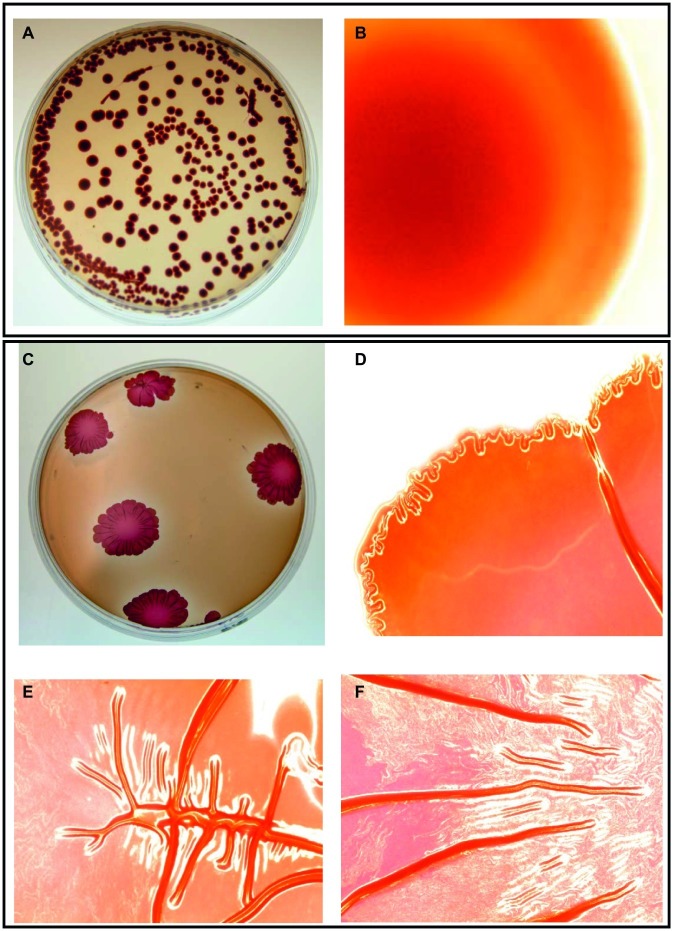
Two distinct morphotypes of *Salmonella* Typhimurium ATCC 14028 growing on LB agar containing Congo red and coomassie brilliant blue at 37°C in 48 h. Smooth morphotype - **(A)** Typical round individual colonies, and **(B)** Round colony edges. Rugose morphotype developed after subinhibitory NaOCl exposure - **(C)** Characteristic red dry and rough (rdar) individual colonies, **(D)** Corrugated colony edges, and **(E,F)** Corrugated colony middle. Microscopic colony images **(B,D,E,F)** were taken using Life Technologies EVOS inverted digital microscope with 4X objective. Similar images were observed for *S.* Heidelberg ATCC 8326.

*Salmonella* cell aggregates and pellicle formation were observed after daily passages for 18 days in the presence of subinhibitory NaOCl in TSB at 37°C. An aliquot of overnight incubated rugose culture in TSB was plated on TSA and characteristic corrugated and dry morphology was formed after 24 h at 37°C. Rugose morphotype was stable at 37 and 25°C and did not revert to smooth morphotype after several subculturing on TSA without NaOCl. Smooth morphotype formed homogenous suspension in TSB, however, rugose colonies were aggregated and elastic even after suspending in TSB. On Congo red agar, smooth morphotype formed smooth edge colonies with no corrugations while rugose morphotype formed characteristics red, dry and rough (rdar) colonies and developed corrugate edged patterns at 37°C after 48 h. Both *S.* Typhimurium ATCC 14028 and *S.* Heidelberg ATCC 8326 formed rdar morphology on Congo red agar (Figure 1).

### *Salmonella* Rugose Morphotype Produced Denser Biofilms on Polystyrene and Stainless Steel Surfaces Than Smooth Morphotype

**Figure 2:**
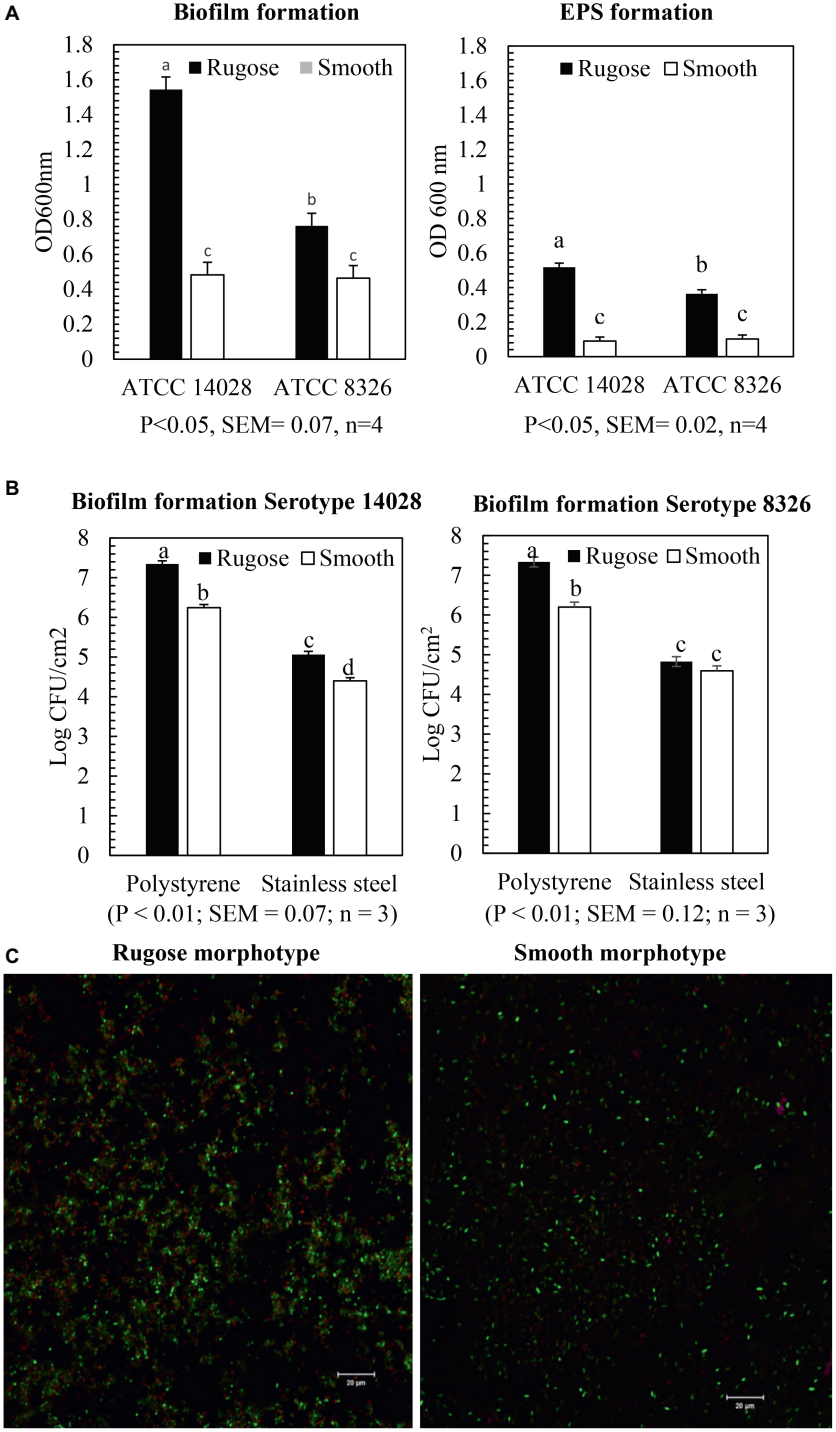
Biofilm and EPS formation of two morphotypes of *S.* Typhimurium (ATCC 14028) and *S.* Heidelberg (ATCC 8326) in 48 h at 25°C. **(A)** Bars represent average OD_600_ value of the biofilm and EPS formation on polystyrene surface after staining with crystal violet assay and ruthenium red assay. The black bar represents rugose morphotype and white bar represents smooth morphotype, **(B)** Bars represent average CFU/cm^2^ value of cellular density of the biofilm formation on polystyrene surface and SS surface. The black bar represents rugose morphotype and white bar represents smooth morphotype, and **(C)** Scanning confocal images of biofilms of *S.* Typhimurium (ATCC 14028) rugose and smooth morphotypes after 48 h at 25°C. Violet color indicates exopolysaccharides, red color indicates protein compounds and green color indicates nucleic acids.

Crystal violet assays showed that rugose morphotype of *S.* Typhimurium ATCC 14028 and *S.* Heidelberg ATCC 8326 produced significantly higher biofilm biomass than the smooth morphotype on polystyrene surface at 25°C (*p* ≤ 0.05) (Figure 2A). The OD_600nm_ for biofilm of rugose morphotype of strains ATCC 14028 and ATCC 8326 were increased by 67 and 29%, respectively, as compared to their corresponding smooth morphotypes (Figure 2A). The CFU enumeration assay showed that rugose morphotype of strain ATCC 14028 produced significantly higher cellular density biofilm than corresponding smooth morphotype on polystyrene and SS surface at 25°C after 48 h. Rugose morphotype of *S.* Typhimurium ATCC 14028 formed biofilm on polystyrene and SS surface with cell density of 7.3 and 5 log CFU/cm^2^ whereas corresponding smooth morphotype formed biofilm on both the surfaces with cell density of 6.2 and 4.2 log CFU/cm^2^. The rugose morphotype of ATCC 8326 produced significant higher cellular density biofilm than corresponding smooth morphotype only on polystyrene surface (*p* ≤ 0.05) where the rugose morphotype formed biofilm with cell density of 7.2 log CFU/cm^2^ and smooth morphotype formed biofilm with cell density of 6.2 log CFU/cm^2^ (Figure 2B). Scanning confocal images of *S.* Typhimurium ATCC 14028 biofilm of rugose morphotype showed more exopolysaccharides (violet color), protein compounds (red color) and nucleic acid (green color) than smooth morphotype (Figure 2C).

### Measurement of Exopolysaccharide Production by Rugose and Smooth Morphotype on Polystyrene Surface

Ruthenium red dye is a carbohydrate binding dye and can detect EPS component of biofilms. The dye is not soluble after it binds to carbohydrate. Thus, EPS component of biofilm was measured by estimating the amount of bound dye after comparing the amount of unbound dye with amount of dye initially added. Ruthenium red assays showed that rugose morphotype of *S.* Typhimurium ATCC 14028 and *S.* Heidelberg ATCC 8326 produced significantly higher EPS compounds after 48 h on the polystyrene surfaces than smooth morphotype (*p* ≤ 0.05) (Figure 2A). EPS production of rugose morphotype of the *S.* Typhimurium ATCC 14028 and *S.* Heidelberg ATCC 8326 was increased by 80 and 71% when compared with their corresponding smooth morphotypes. EPS staining of the biofilm wells show that rugose morphotype produced a dense biofilm at the air-water interphase as compared to the smooth morphotype (Supplementary Figure S1). EPS staining showed that biofilm formation pattern of rugose morphotypes varied between two strains. Rugose morphotype of ATCC 14028 produced biofilm on air-water interphase and walls of the well, however, ATCC 8326 rugose morphotype formed biofilm predominantly on air-water interphase.

### Effect of Sodium Hypochlorite Subinhibitory Concentration on Rugose and Smooth Morphotype Biofilm Formation Is Strain Dependent

**Figure 3:**
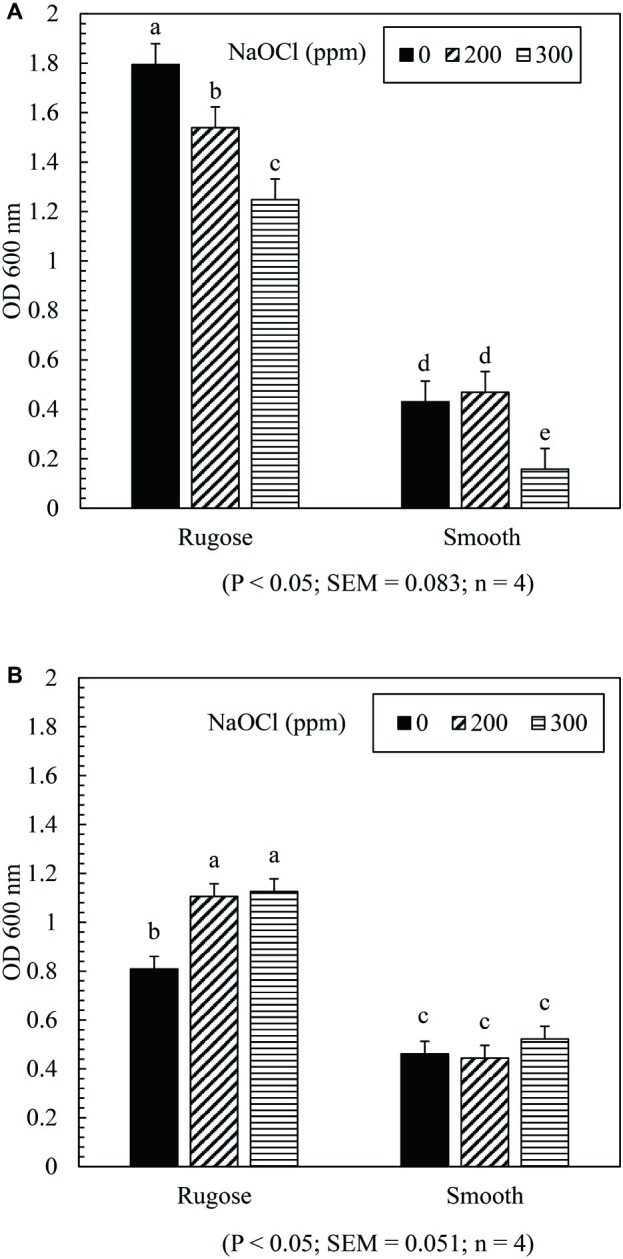
Effect of subinhibitory concentrations (SICs) of NaOCl on biofilm formation of rugose and smooth morphotypes of *S.* Typhimurium ATCC 14028 **(A)** and *S.* Heidelberg ATCC 8326 **(B)**. Bars represent average OD_600_ value of biofilm formation of *S.* Typhimurium and *S.* Heidelberg after staining with crystal violet. The black, diagonal and horizontal bar represents biofilm formation of negative control (without NaOCl exposure), and in the presence of SICs of 200 or 300 ppm NaOCl in TSB of rugose and smooth morphotype, respectively.

The SICs of NaOCl can significantly affect biofilm formation ability of both rugose and smooth morphotypes depending on the tested strain. Biofilm formation of rugose morphotype of *S.* Typhimurium ATCC 14028 reduced significantly in the presence of SICs (200 and 300 ppm) of NaOCl (*p* ≤ 0.05), while the smooth morphotype formed significantly lower biofilm at 300 ppm but not at 200 ppm (*p* ≤ 0.05) (Figure 3A). For *S.* Heidelberg ATCC 8326, rugose morphotype biofilm production increased significantly at SICs (200 ppm and 300 ppm) of NaOCl (*p* ≤ 0.05), while these SICs did not affect biofilm formation ability of smooth morphotype (Figure 3B).

### Inactivation of *Salmonella* Rugose and Smooth Morphotype Biofilms on Polystyrene Surface by Lethal Sodium Hypochlorite

**Figure 4:**
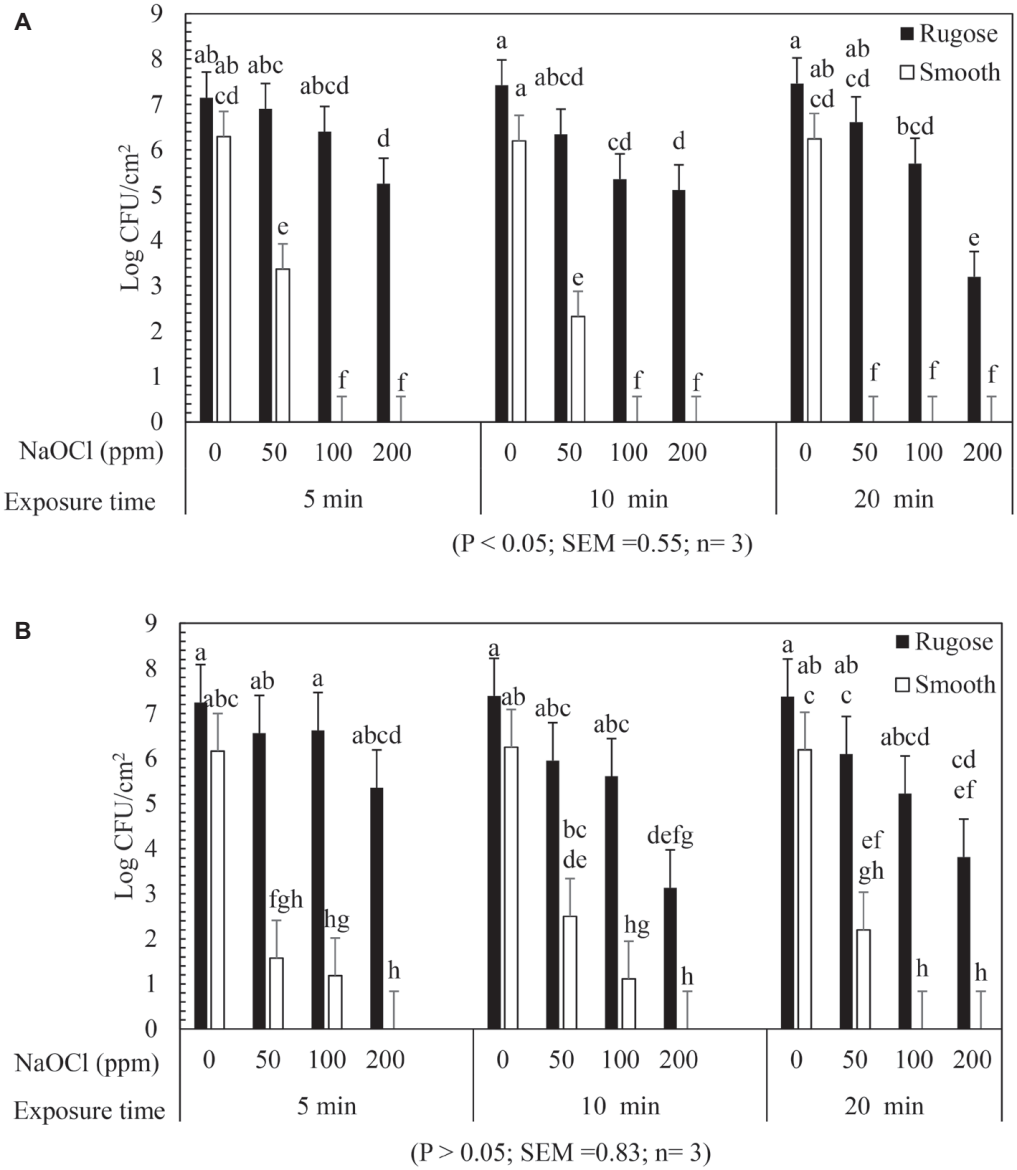
Biofilm survival of *S. Typhimurium* ATCC 14028 and *S. Heidelberg* ATCC 8326 morphotypes on polystyrene surface after NaOCl treatment. Bars represents *S. Typhimurium* **(A)** and *S. Heidelberg* **(B)** biofilm survival at different NaOCl concentrations (50, 100, or 200 ppm in water) for different exposure times (5, 10, or 20 min). In **(A)**, three way significant interaction of factors was found for NaOCl concentrations, exposure time intervals and *Salmonella* morphotypes. In **(B)**, no significant interaction of factors found for NaOCl concentrations, exposure time and *Salmonella* morphotypes.

The inactivating efficacy of NaOCl against preformed *S.* Typhimurium ATCC 14028 and *S.* Heidelberg ATCC 8326 48 h old biofilms on 96-well polystyrene microtiter plates at 25°C is presented in Figure 4. For biofilm inactivation study, three factors treatment combination of: (1) *Salmonella* morphotypes, (2) NaOCl concentrations, and (3) exposure time was investigated. A significant three-factor interaction (NaOCl concentrations × exposure time × morphotypes) was found for *S*. Typhimurium ATCC 14028 (Figure 4A) but not for *S.* Heidelberg ATCC 8326 (Figure 4B). For *S.* Typhimurium ATCC 14028, 2–3 logs of smooth morphotype biofilm cells survived after 50 ppm of NaOCl treatment for 5 or 10 min, and then undetectable after 100 or 200 ppm NaOCl treatment regardless of exposure time (5, 10, or 20 min). By contrast, 3–4 logs of rugose biofilm cells survived even after 200 ppm NaOCl treatment for 20 min on polystyrene surface (Figure 4A; Supplementary Figure S2A).

Similarly, *S. Heidelberg* ATCC 8326 smooth morphotype biofilm cells survived by 2–3 logs after 50 ppm NaOCl treatment, or by 1 log after 100 ppm NaOCl for 5 or 10 min, and were then non-detectable after 200 ppm NaOCl regardless of exposure time. By contrast, the rugose morphotype biofilm cells of S. Heidelberg ATCC 8326 had higher tolerance where 3 logs survived even after 200 ppm NaOCl treatment for 20 min (Figure 4B).

A significant two-factor interaction (NaOCl concentrations × morphotype) was found for *S.* Heidelberg ATCC 8326 (Supplementary Figure S2). Biofilm of *S*. Heidelberg smooth morphotype was below detection limit after 5 min at 100 or 200 ppm NaOCl concentration. However, the rugose morphotype biofilm cells were tolerant to lethal concentrations of NaOCl where as high as 4 logs survived even after 200 ppm NaOCl treatment (Supplementary Figure S2B).

### Inactivation of *Salmonella* Rugose and Smooth Morphotype Biofilms on Stainless Steel Surface by Lethal Sodium Hypochlorite

**Figure 5:**
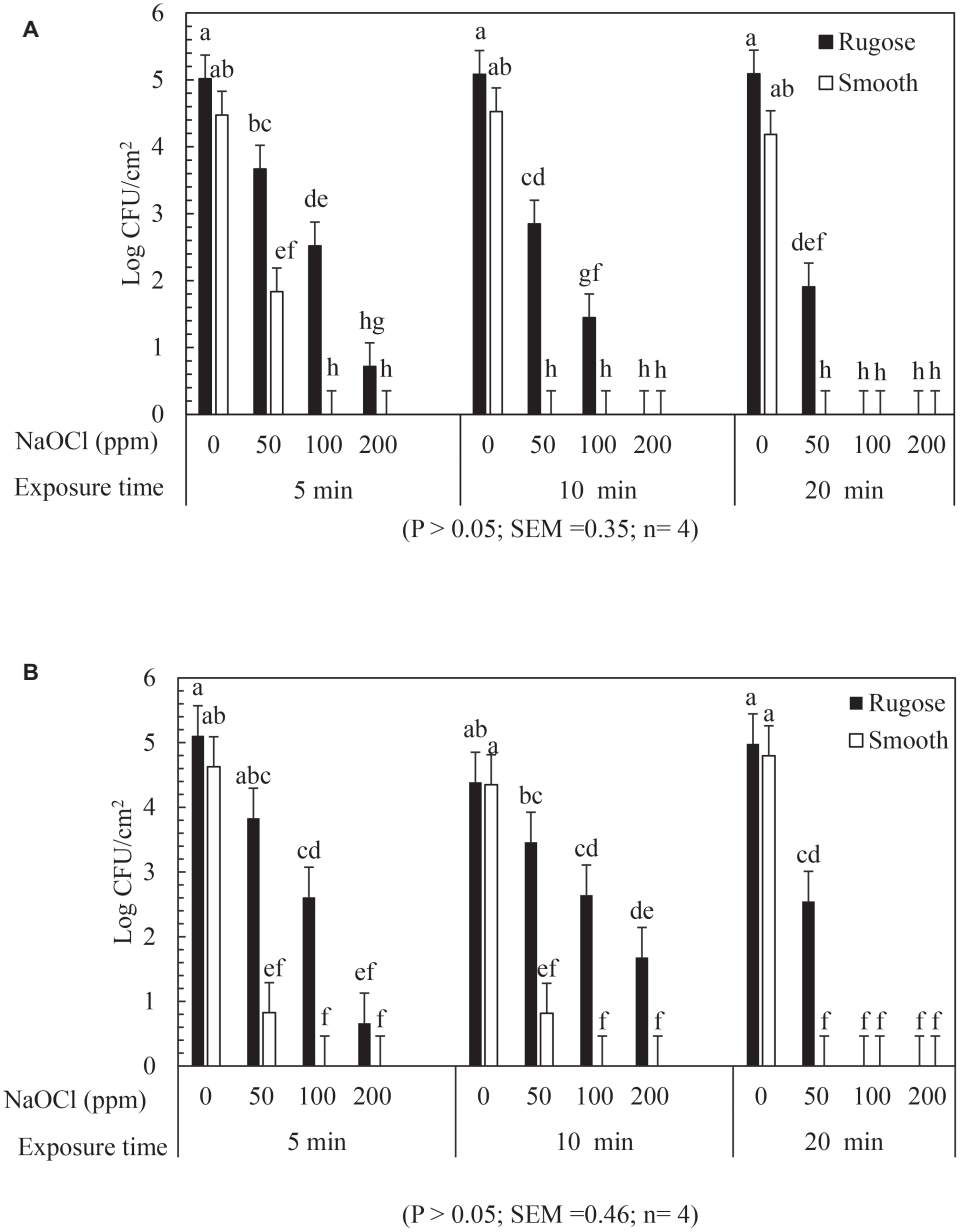
Biofilm survival of *S.* Typhimurium ATCC 14028 and *S.* Heidelberg ATCC 8326 morphotypes on stainless steel surface after NaOCl treatment. Bars represents S. Typhimurium **(A)** and S. Heidelberg **(B)** biofilm survival at different NaOCl concentrations (50, 100 or 200 ppm in water) for different exposure times (5, 10, or 20 min).

The inactivating efficacy of NaOCl against *S.* Typhimurium ATCC 14028 and *S.* Heidelberg ATCC 8326 biofilms on SS coupons at 25°C is presented in Figure 5 for three-factor interactions. For *S*. Typhimurium ATCC 14028, 2 logs of smooth morphotype biofilm cells survived after 50 ppm NaOCl for 5 min, and were then non-detectable after 100 or 200 ppm NaOCl regardless of exposure times. By contrast, the rugose morphotype biofilm cells of *S.* Typhimurium ATCC 14028 survived by 2–3 logs after 50 ppm NaOCl treatment at all exposure times, and by 1–2 logs survival after 5 or 10 min exposure to 100 ppm NaOCl, and were then non-detectable at 200 ppm of NaOCl (Figure 5A).

Similarly, *S.* Heidelberg ATCC 8326 smooth morphotype biofilm cells recovered by 1–2 logs after 50 ppm NaOCl for 5 or 10 min, and then non-detectable after 100 and 200 ppm of NaOCl. By contrast, the rugose morphotype biofilm cells were tolerant to 100 ppm NaOCl where 1–2 logs survived even after 20 min exposure time but not at 200 ppm of NaOCl (Figure 5B).

A significant two-factor interaction effect (morphotype × NaOCl concentration) was found for biofilm inactivation of *S*. Typhimurium ATCC 14028 and *S*. Heidelberg ATCC 8326 on SS surface. Biofilm cells of smooth morphotype of ATCC 14028 and 8,326 were non-detectable on SS surface at 100 or 200 ppm NaOCl for 5 min. However, the rugose biofilm cells were recovered by 0.3 log for ATCC 14028 and by 1 log for ATCC 8326 even after 200 ppm NaOCl (Supplementary Figures S3A,B).

### Gene Expression for Biofilm Formation and Multicellular Behavior in *Salmonella* Typhimurium

Table 2 shows the critical gene expression levels for biofilm formation and rugose development comparing smooth and rugose morphotypes. Sequential exposure of sodium hypochlorite at SICs increased the expression of *envZ*, *csgD*, *csgA*, *csgB*, *csgC*, *bcsE*, *hns*, *rpos,* and *csrA* by ∼ 139.5, 100.3, 2.84, 11.03, 11.3, 2.35, 3.06, 6.36, and 5.67 fold, respectively (Table 2). However, there was no significant difference in gene expression for fimbrin, flagellin, outer membrane secretin, and *csgD* regulator i.e. *ompR* and *ihf* between smooth and rugose morphotypes.

**Table 2:** Fold change in the expression of *S*. Typhimurium ATCC 14028 biofilm and multicellular behavior associated genes.

Gene	Gene product or function	Fold change to 16S rRNA	
		Smooth morphotype	Rugose morphotype
*envZ*	Receive environmental signals and phosphorylates *ompR*	1.36 ± 0.40	**139.5 ± 72.9** *
*spiA*	Outer membrane secretin	0.90 ± 0.07	0.87 ± 0.14
*bcsE*	Cellulose synthesis	0.73 ± 0.13	**2.35 ± 0.54** *
*csgD*	Curli and cellulose compounds formation	1.01 ± 0.05	**100.3 ± 26.4** *
*ycfR*	Stress responses and biofilm formation	1.33 ± 0.49	1.17 ± 0.36
*csgA*	Curlin major subunit	0.89 ± 0.10	**2.84 ± 0.88** *
*csgB*	Surface exposed nucleator for *CsgA*	1.02 ± 0.07	**11.03 ± 2.42** *
*csgC*	Putative curli production protein	1.01 ± 0.07	**11.13 ± 3.52** *
*hns*	*csgD* transcription	1.08 ± 0.17	**3.06 ± 0.62** *
*rpoS*	Stress resistance and biofilm formation	1.02 ± 0.09	**6.36 ± 1.72** *
*ompR*	*csgD* transcription	1.02 ± 0.07	1.10 ± 0.12
*ihf*	*csgD* transcription	1.00 ± 0.03	1.11 ± 0.20
*fimA*	Major type 1 subunit fimbrin (pilin)	0.91 ± 0.12	1.28 ± 0.20
*fimH*	Binding with eukaryotic host cells	1.01 ± 0.06	2.23 ± 0.52
*fliC*	Filaments of bacterial flagella	1.02 ± 0.07	2.00 ± 0.35
*csrA*	Metabolism, virulence, and stress responses	1.02 ± 0.06	**5.67 ± 0.75** *

*Real time quantitative PCR was conducted with 16S rRNA serving as endogenous control*.

* *Significant change in the expression of genes at *p* ≤ 0.05 are highlighted in bold*.

### Scanning Electronic Microscopy of Rugose and Smooth Morphotype Biofilms on Polystyrene and Stainless Steel Surfaces

**Figure 6:**
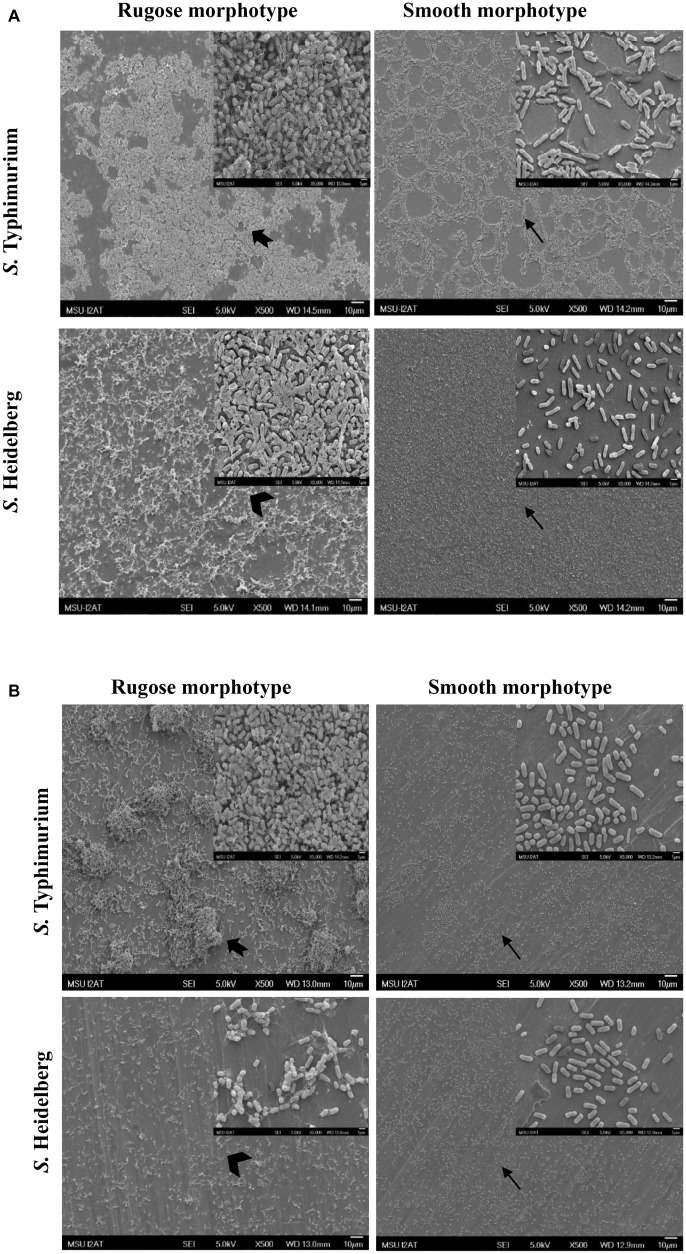
Scanning electron micrographs of biofilms of *S.* Typhimurium and *S.* Heidelberg morphotypes on: **(A)** polystyrene surface, and **(B)** SS surface after 48 h at 25°C. Notched arrow indicate thick aggregates rugose biofilm of *S.* Typhimurium while arrow head indicate sparse aggregates of rugose biofilm of *S.* Heidelberg. Arrows indicate monolayer of smooth morphotype biofilm of both the strains.

Structural differences in rugose and smooth morphotype biofilms were investigated using scanning electron microscopy. Rugose morphotype formed thick aggregates of cells surrounded with higher amount of EPS on both polystyrene and SS surfaces while smooth morphotype formed a monolayer of biofilm under the same conditions. On polystyrene surface, rugose morphotype of *S.* Typhimurium ATCC 14028 formed thick 3D biofilm while *S.* Heidelberg ATCC 8326 formed thick macro-colonies whereas smooth morphotype formed thin monolayer of cells (Figure 6A). On SS surface, rugose morphotype of ATCC 14028 formed thick macro colonies of biofilms while ATCC 8326 formed thick aggregated but sparse colonies whereas smooth morphotype formed thin monolayer of cells (Figure 6B). Also, at higher magnification, SEM micrographs revealed a majority of rugose morphotype cells were interconnected with extracellular material while smooth morphotype cells were separated from each other for both strains.

### Transmission Electron Microscopy of Rugose and Smooth Morphotypes

**Figure 7:**
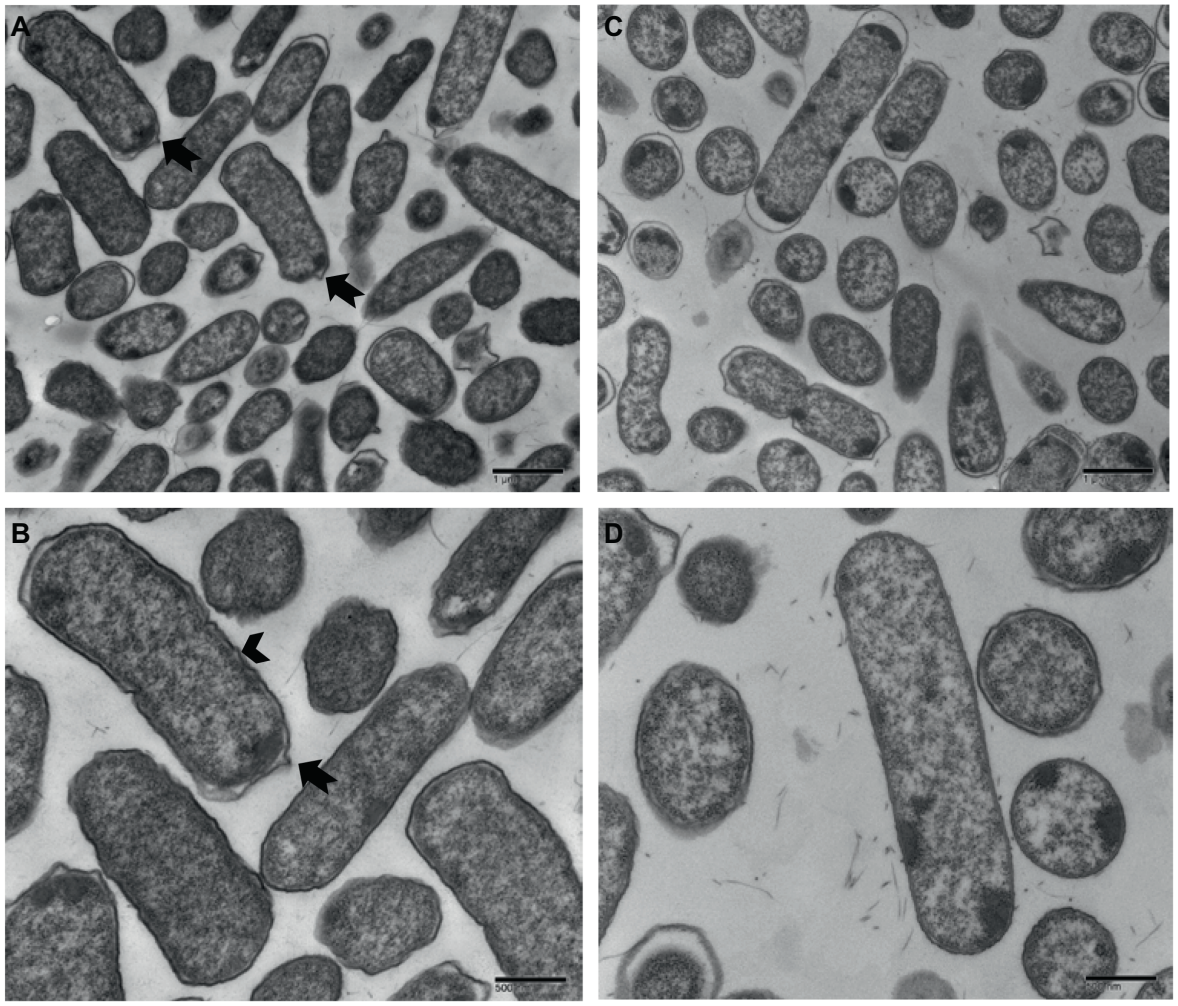
Transmission electron micrographs of *Salmonella* Typhimurium ATCC 14028 rugose morphotype cells at different magnifications **(A,B)** and smooth morphotype cells at different magnifications **(C,D)**. Notched arrow indicates membrane vesicle formation and arrowhead indicate rough and thick cell wall.

Ultrastructural variation in planktonic cells of smooth and rugose morphotypes for *S.* Typhimurium ATCC 14028 was investigated using transmission electron microscope. The cell wall of most planktonic smooth cells was smooth without any corrugations; however, the rugose cell wall was thickened, rough with bleb formations. The thickness of rugose cell wall could be attributed to higher extracellular polymeric compounds which is hallmark of biofilm formation (Figure 7).

## Discussion

The MIC of NaOCl for *S.* Typhimurium ATCC 14028 and *S.* Heidelberg ATCC 8326 was determined to be 400 ppm for smooth morphotype and 500 ppm for rugose morphotype in TSB. Therefore, the concentrations of 200 and 300 ppm of NaOCl were subinhibitory in TSB for both morphotypes of these two serovars. TSB has an organic content which interacts with NaOCl, however, water does not have organic content. Hence, the biocidal concentration of NaOCl was about 10× higher in broth versus water. Due to the higher availability of NaOCl in water, it has a biofilm inactivating effect at even low concentration of 50 ppm. Thus, the NaOCl concentrations of 50, 100, or 200 ppm were used in water for biofilm inactivation study.

As we have reported previously (Obe et al., 2018), the sequential exposure to progressively increasing subinhibitory concentrations of NaOCl in TSB at 37°C led to the development of stable rugosity in both *S.* Typhimurium ATCC 14028 and *S.* Heidelberg ATCC 8326. Then the stable behavior of rugose morphotype was confirmed by frequent subculturing of rugose *Salmonella* on TSA and TSB without NaOCl. These results showed that *Salmonella* rugose is a stable morphotype and does not revert to smooth morphotype even after culturing conditions with absence of the factor which had induced rugosity. In other studies, *Salmonella* DT 104 also developed rugosity in 4 days at 19–28°C on TSA (Anriany et al., 2001). Other factors that contribute to rugose morphotype development in *Salmonella* are: (1) temperature stress (Gerstel and Romling, 2001), (2) pH (Romling et al., 1998), and (3) osmolarity (Romling et al., 1998; Prigent-Combaret et al., 2001). Anriany et al., 2001 reported *Salmonella* DT 104 forms rugose colonies on nutrient rich medium with low osmolarity at 25°C but not at 37°C. However, in the present study, both the strains (*S.* Typhimurium and *S.* Heidelberg) formed rugose after NaOCl adaptation at 25 and 37°C in nutrient rich medium. In another study 4% ethanol in growth medium induced higher transcription of *csgD* resulted in formation of rugose during logarithmic growth phase. Similarly, microaerophilic conditions in nutrient rich medium (LB without salt) induced higher tendency to form rugose compared to aerobic and anerobic conditions (Gerstel and Römling, 2003).

Apart from laboratory conditions, *Salmonella* rugose strains were also isolated from meat sources. Previous finding shows that the *Salmonella* rugose morphotype is commonly prevalent (56.4%) in strains isolated from turkey meat (Karaca et al., 2013). *Salmonella* rugose development is associated with a higher formation of thin, aggregative fimbriae also known as curli and cellulose compounds (Gerstel and Römling, 2003) which could be associated with higher expression of biofilm regulator *csgD*. Curli are the major proteinaceous component of complex EPC(s) produced by *Salmonella* rugose morphotype. In the present study, the curli formation by *Salmonella* rugose was determined by culturing cells on Congo red agar after 48 h at 37°C. The β strands of curli subunits of rugose morphotype binds with hydrophobic Congo red dye to form red dry and rough (rdar) colonies, however, in the absence of curli formation smooth morphotype produced smooth edge colonies on Congo red agar (Figure 1).

Curli and cellulose are two major EPC(s) of *Salmonella* biofilm. Overexpression of curli and cellulose reinforce *Salmonella* rugose morphotype biofilm formation on abiotic surfaces. The *Salmonella* rugose and smooth morphotype biofilm formation was frequently assessed by the crystal violet (CV) assay and CFU enumeration method. The crystal violet assay is the standard, simple and high throughput method to assess biofilm formation to an abiotic surface such as polystyrene or polypropylene and measures all components of biofilm such as EPC(s) and cellular content. The CFU of biofilm on TSA was used to enumerate viable, strongly attached biofilm cells on polystyrene and SS surface. In poultry abattoir, the temperature in reception and evisceration area is around 25°C and also there was higher biofilm formation by *Salmonella* at ambient temperature (Soni et al., 2013). Therefore, *Salmonella* rugose biofilm formation ability was investigated at ambient temperature of 25°C in the present study. The CV assay revealed that the rugose morphotypes of *S.* Typhimurium ATCC 14028 and *S*. Heidelberg ATCC 8326 produced dense biofilm on polystyrene surface (Figure 2A). Rugose dense biofilm formation ability is associated with increased attached biofilm cells and higher formation of EPC (s). Further, the CFU enumeration method showed that biofilm of rugose morphotype of *S.* Typhimurium ATCC 14028 had significantly higher attached cell concentration on polystyrene and SS surfaces, while for rugose morphotype of *S*. Heidelberg ATCC 8326 had significantly higher attached cell concentration only on polystyrene surface (Figure 2B). These results showed that rugose morphotype biofilm forming ability differs depending on strains and the surfaces.

Subsequently, the actual EPS production in biofilm was estimated by ruthenium red assay which measures carbohydrate dye binding ability for two morphotypes. The ruthenium red assay showed rugose morphotype biofilm produced significant higher amount of carbohydrate than smooth morphotype. Confocal scanning micrographs validated the findings from CV and ruthenium red assay that rugose cells are in a dense aggregate form as compared to the monolayer of smooth morphotype (Figure 2C). The results showed that increase in biofilm formation for rugose morphotype could be the result of both overexpression of EPC(s) and increase in the number of strongly attached cells to the surfaces.

Stress adaptation induced rugose morphotype forms higher EPC(s) and stronger biofilm which may help *Salmonella* to survive in the food processing environmental conditions (Steenackers et al., 2012). In the food processing facility, *Salmonella* biofilm is regularly exposed to sublethal stresses. The results showed that exposure to subinhibitory concentrations of NaOCl (200 or 300 ppm in TSB) did not influence the biofilm formation ability of the smooth morphotype of *S.* Heidelberg ATCC 8326 but at 300 ppm exposure, biofilm formation was decreased for *S.* Typhimurium ATCC 14028. The rugose morphotype biofilm formation ability was influenced by the presence of subinhibitory concentration of NaOCl (200 or 300 ppm in TSB) in an opposite manner between the two strains. Thus, *S.* Heidelberg ATCC 8326 biofilm formation ability significantly increased and *S.* Typhimurium ATCC 14028 biofilm formation ability was significantly decreased (Figures 3A,B). In a previous study of *S.* Typhimurium strain (S175), there was enhancement of biofilm formation after contact with subinhibitory concentrations of NaOCl (Capita et al., 2017). However, in another study, disruption of quorum sensing by sublethal carvacrol reduced *S*. *Typhi* biofilm formation ability. Thus, the variability in the biofilm formation may be influenced by multiple factors such as bacterial adaptation, expression of biofilm related genes, quorum sensing process or strain variation. The tendency of the *S.* Heidelberg rugose morphotype to form denser biofilm under sublethal stress condition may increase its ability to persist in the food processing environment.

The ability of the rugose morphotype to produce more EPC(s) such as curli and cellulose may provide resistance of its biofilm form against antimicrobials such as NaOCl (White et al., 2006). To investigate the NaOCl tolerance in rugose biofilm at ambient temperature conditions, the 48 h old *Salmonella* biofilm grown at 25°C was exposed to three different NaOCl concentrations (50, 100, or 200 ppm) in water for three different time intervals (5, 10, or 20 min) at pH 6.15. In a previous study, the exposure time of 10–15 min at 100–200 ppm NaOCl was used for *Salmonella* biofilm inactivation (Iñiguez-Moreno et al., 2018). Our results show that rugose morphotype biofilm had increased tolerance against treatment of NaOCl on polystyrene and SS surfaces at room temperature (Figures 4, 5). The increased tolerance of rugose morphotype against treatment of NaOCl could be attributed to higher EPC(s) production. The EPC(s) are organic compounds which can neutralize the antimicrobial activity of NaOCl. The scanning electron micrographs revealed that rugose biofilm cells of ATCC 14028 and ATCC 8326 were densely imbedded in EPC(s) on both polystyrene and SS surfaces. However, the rugose biofilm cells on the polystyrene surface were present in thick layers, while these were present in thick aggregated microcolonies on the SS surface (Figure 6).

Previous studies indicate that SICs of plant derived antimicrobials changes the expression of biofilm related gene expression of *Listeria monocytogenes* (Upadhyay et al., 2013). In the present study, the sequential exposure of sodium hypochlorite at SICs modulated the rugose and biofilm related gene expression levels in *S.* Typhimurium.

Other studies show that the major component of *S*. Typhimurium biofilm, i.e., curli and cellulose formation is co-regulated by biofilm regulator (*csgD*). The gene expression data in our study indicate that the expression of csgD reguhlator (hns), biofilm regulator (*csgD*), curli synthesis (*csgA, csgB*, and *csgC*) and cellulose formation (*bcsE*) was significantly increased in rugose morphotype when induced by sequential exposure of sodium hypochlorite SICs. Sodium hypochlorite induced oxidative stress in *S*. Typhimurium may have resulted in higher expression of environment stress related genes such as *envZ* and *csrA* (Table 2). However, there was no significant change in the expression levels of the biofilm related genes of fimbriae and filament in these conditions.

When observed under transmission electron microscope, *Salmonella* rugose planktonic cells have rough and thickened cell wall and more cell surface area compared to the smooth cells. Planktonic rugose cell wall had surrounding EPS material where smooth cells did not have any such material (Figure 7). Also, membrane vesicle formation is associated with rugose planktonic cells. Previous studies show that thickened cell wall (To et al., 2002), and outer membranous bleb development (Kulp and Kuehn, 2010) is associated with resistance against antimicrobials in bacterial cells. Transmission electron micrographs in the current study showed that rugose cells have a thickened and rough cell wall and outer membranous bleb which may contribute in antimicrobial tolerance to NaOCl.

These findings show that *Salmonella* rugose, a multicellular morphotype characterized by formation of cellulose, curli and by production of EPC(s) may aid in its survival and persistence in the food processing environments, thereby increase its transmission between surfaces and hosts. Exposure to subinhibitory NaOCl may enhance *Salmonella* rugose morphotype biofilm formation. Also, the higher curli and cellulose formation in rugose morphotype protects its biofilm which provides an increased tolerance and survival against disinfectants such as NaOCl.

## Data Availability Statement

The raw data supporting the conclusions of this manuscript will be made available by the authors, without undue reservation, to any qualified researcher.

## Author Contributions

The research was designed and led by RN from FSNHP and by CS, CM, and AK from POSC and was completed by MB and DK. SC contributed to reagents and materials for the gene expression study.

### Conflict of Interest

The authors declare that the research was conducted in the absence of any commercial or financial relationships that could be construed as a potential conflict of interest.
