# Supplementary material for: Rugose Morphotype in Salmonella Typhimurium and Salmonella Heidelberg Induced by Sequential Exposure to Subinhibitory Sodium Hypochlorite Aids in Biofilm Tolerance to Lethal Sodium Hypochlorite on Polystyrene and Stainless Steel Surfaces
Source: Front Microbiol. 2019 Nov 27;10:2704. doi: 10.3389/fmicb.2019.02704 (PMC6890808; doi:10.3389/fmicb.2019.02704)
Supplement: Supplementary file 1 [file Image_1.pdf]

Figure S1

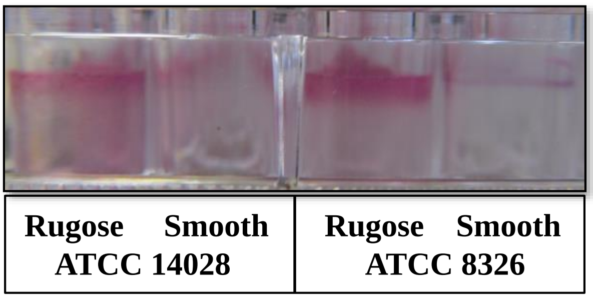

Figure S1 | Ruthenium red staining of biofilm on polystyrene surface of *S.* Typhimurium ATCC 14028 and S. Heidelberg 8326 Rugose morphotype cells produces dense biofilm (pink color) compare to smooth morphotype
