# Supplementary material for: Rugose Morphotype in Salmonella Typhimurium and Salmonella Heidelberg Induced by Sequential Exposure to Subinhibitory Sodium Hypochlorite Aids in Biofilm Tolerance to Lethal Sodium Hypochlorite on Polystyrene and Stainless Steel Surfaces
Source: Front Microbiol. 2019 Nov 27;10:2704. doi: 10.3389/fmicb.2019.02704 (PMC6890808; doi:10.3389/fmicb.2019.02704)
Supplement: Supplementary file 2 [file Image_2.pdf]

Figure S2

(A)

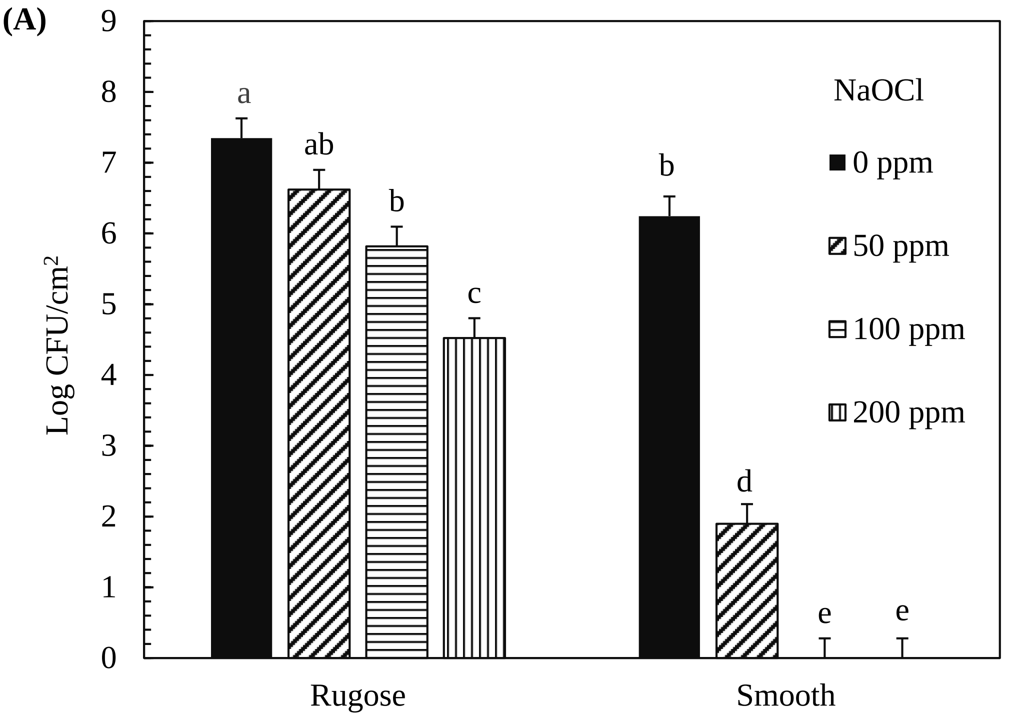

(P < 0.05; SEM = 0.27; n = 3)

(B)

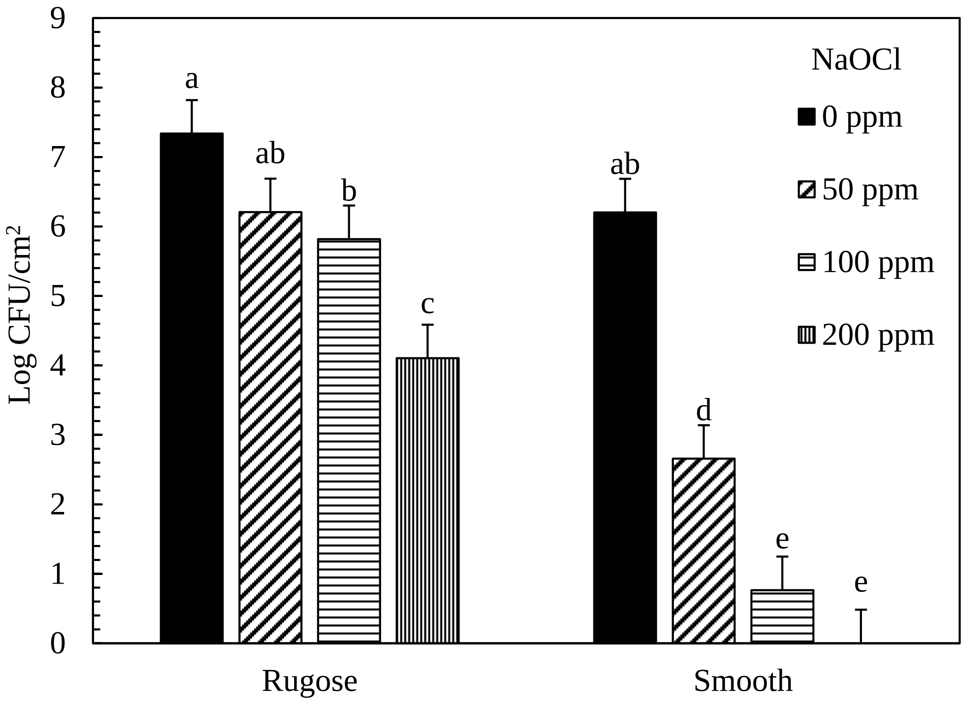

(P < 0.05; SEM = 0.48; n = 3)

Figure S2 │ Biofilm survival of *S.* Typhimurium ATCC 14028 and *S.* Heidelberg ATCC 8326 morphotypes on polystyrene surface after NaOCl treatment. Bars represent *S.* Typhimurium (A) and *S.* Heidelberg (B) biofilm survival at different NaOCl concentration (50, 100 or 200 ppm in water). In the figure two way significant interaction of factors found for NaOCl concentrations and *Salmonella* morphotypes.
